# Thermal and Radiation Stability in Nanocrystalline Cu

**DOI:** 10.3390/nano13071211

**Published:** 2023-03-29

**Authors:** Marie Thomas, Heather Salvador, Trevor Clark, Eric Lang, Khalid Hattar, Suveen Mathaudhu

**Affiliations:** 1 Metallurgical and Materials Engineering Department, Colorado School of Mines, Golden, CO 80401, USA; 2 Mechanical Engineering Department, University of California, Riverside, CA 92521, USA; 3 Materials Science and Engineering Program, University of California, Riverside, CA 92521, USA; 4 Center for Integrated Nanotechnologies, Sandia National Laboratories, Albuquerque, NM 87185, USA; 5 Department of Nuclear Engineering, University of New Mexico, Albuquerque, NM 87131, USA; 6 Department of Nuclear Engineering, University of Tennessee, Knoxville, TN 37996, USA

**Keywords:** copper, irradiation, nanocrystalline, stability

## Abstract

Nanocrystalline metals have presented intriguing possibilities for use in radiation environments due to their high grain boundary volume, serving as enhanced irradiation-induced defect sinks. Their promise has been lessened due to the propensity for nanocrystalline metals to suffer deleterious grain growth from combinations of irradiation and/or elevated homologous temperature. While approaches for stabilizing such materials against grain growth are the subject of current research, there is still a lack of central knowledge on the irradiation–grain boundary interactions in pure metals despite many studies on the same. Due to the breadth of available reports, we have critically reviewed studies on irradiation and thermal stability in pure, nanocrystalline copper (Cu) as a model FCC material, and on a few dilute Cu-based alloys. Our study has shown that, viewed collectively, there are large differences in interpretation of irradiation–grain boundary interactions, primarily due to a wide range of irradiation environments and variability in materials processing. We discuss the sources of these differences and analyses herein. Then, with the goal of gaining a more overarching mechanistic understanding of grain size stability in pure materials under irradiation, we provide several key recommendations for making meaningful evaluations across materials with different processing and under variable irradiation conditions.

## 1. Introduction

During irradiation, atomic displacements within a metallic lattice result in a variety of microstructural changes, such as dislocation loop and network formation, stacking fault tetrahedra (in FCC metals), precipitation, partitioning, and void formation [1,2,3]. These changes affect the material properties and eventually lead to material failure [3,4]. For example, exposing metals to irradiation results in hardening and embrittlement due to the production of defects which impede the motion of dislocations [3,5,6] and degrade the thermal conductivity [7].

The resistance of a material to radiation damage is determined by its ability to accommodate radiation-induced point defects (vacancies and interstitials) [8]. Radiation damage tolerance can be enhanced by controlling the point defect mobility. One approach for controlling defect mobility is via chemical stabilization, for example, through alloying additions. Mao et al. demonstrated that adding W to Cu increases the migration energy for vacancy and the threshold displacement energy, leading to lower point defect diffusivity [9]. Another approach of limiting defect mobility is through the introduction of point, planar, or volumetric defect sinks, such as grain boundaries, phase boundaries, twin boundaries, nanopores, nanoparticles, and nanoclusters, as trapping sites [1,2,10,11,12].

The sink efficiency of these microstructural features has been used to describe the ability of an interface to reduce radiation damage by absorbing nearby defects [1,13,14,15]. It is defined as the ratio of defects absorbed by a boundary to defects absorbed by a perfect sink [14,16]. Sink strength describes the effect of defect sinks spread throughout the material [15]. Evaluating and comparing sink efficiencies and strengths is key to designing radiation tolerant materials [17]; however, they are experimentally challenging to measure. Sink efficiency has been quantified by measuring the defect denuded zones [18], but denuded zones are not always observed [14]. Their presence is a consequence of defect trapping at the interfaces, and in the case of grain boundaries it may vary depending on the grain boundary character [19,20,21,22,23], the strength of other sinks [13,24], grain size [9], defect recombination rate [13,14], and irradiation conditions [9,14,22].

Nanostructured materials have spurred interest due to the increased grain boundary volume with decreasing grain size [25], and therefore have been identified as promising candidates for radiation-tolerant materials due to their high sink density [9,26]. This benefit is offset by the propensity for nanocrystalline materials to suffer from detrimental grain growth at low homologous temperatures [27,28,29]. Nanocrystalline metals without kinetic stabilization tend to undergo grain growth to minimize the high grain boundary energy present. As such, they are not thermodynamically stable and can lose their ability to tolerate damage during prolonged irradiation. Grain growth during irradiation has been observed in various materials even at low temperatures where no thermally induced grain growth would be expected [30,31,32,33,34,35,36,37]. Further, at higher homologous temperatures this irradiation-induced grain growth couples with thermally driven grain growth.

In response, efforts have been made to design materials with a combination of various defect sinks to enhance both thermodynamic and radiation stabilities. For example, nanotwinned Cu with nanovoids has been studied under ion irradiations and has shown good damage tolerance and better thermal stability than nanocrystalline Cu [38,39]. However, more widespread progress has been limited by the lack of a knowledge of the fundamental interaction mechanisms between radiation-induced defects and grain boundary sinks, which is needed to quantify the performance of unalloyed nanocrystalline materials as a function of irradiation conditions. This lack of collective understanding can be in part explained by the large variations of study parameters complicating a comparison of results combined with the lack of in-situ/operando experimental capabilities.

This review paper seeks to unravel the extant literature on the defect stability of nanocrystalline primarily FCC materials through an examination of unalloyed Cu as a model system, and further explorations of a handful of dilute Cu alloys. This system is chosen based on the breadth of literature across grain size regime, processing conditions, irradiation conditions, and temperatures. Further, these conditions have led to complexity in analyses and often incongruent reported findings. Additionally, pure metals are desirable model systems to study radiation damage as there is no influence of secondary phases [2]. The behaviors of Cu under irradiation conditions are certainly not representative of all materials classes, and therefore this review focuses primarily on how the variation of theoretical and experimental analyses can lead to a lack of cohesive knowledge on Cu, which would have a similar affect in other materials classes. With a more holistic picture of the span of the literature, we propose some guidelines for the generation of consistent, valid information and conclusions in this space.

The structure of this paper will sequentially review the basis of the literature for pure Cu. In Section 2, how grain size affects the radiation damage tolerance will be summarized. Section 3 focuses on the grain growth regimes in irradiation and/or thermal environments. In Section 4, the implications of synthesis and processing on grain boundary character and thus the response to irradiation are discussed. The effect of irradiation on material properties as a function of grain size is presented in Section 5. In Section 6, the impact of the radiation environment and why it is critical to consider it while comparing and analyzing data is addressed. Finally, Section 7 will discuss the impact of the collective findings from the literature on factors that cloud conclusive observations and findings.

## 2. Grain Size Impact on Stability

With grain boundaries serving as effective defect sinks, one can wonder if a smaller grain size always results in higher damage tolerance due to higher sink density or if there is a limit. Numerous studies have shown enhanced radiation damage tolerance in nanocrystalline materials [4,22,23,40]. Lower defect densities have been measured in nanocrystalline (NC) Cu compared to coarse-grained (CG) Cu after He ion bombardment at high temperatures [9,22]. Similar results are reported for NC-Ni after in situ ion irradiation [40]. Room-temperature Kr ion irradiation of NC-Pd demonstrates a decrease in defect density as the grain size decreases from 80 to 10 nm [23]. Improved radiation tolerance in nanocrystalline materials has also been measured in terms of defect size (i.e., vacancy loops and stacking fault tetrahedra). An increase in cavity size with grain size was observed in NC-Cu under ion irradiation [22]. Similarly, Barr et al. reported an increase in the maximum size of dislocation loops with increasing grain size in the range 20–100 nm in NC-Pt. They ascribed this increase to the ability of dislocation loops to grow and coalesce inside larger grains [18].

Using molecular dynamics (MD) simulations, Bai et al. explained this enhanced radiation tolerance by a “loading-unloading” mechanism. In this proposed mechanism, interstitials migrate to the grain boundaries and are absorbed in them. The interstitial-loaded grain boundaries then emit interstitials to annihilate bulk vacancies. This recombination mechanism has a lower energy barrier than vacancy diffusion, allowing for the removal of less-mobile vacancies [41]. Other mechanisms have been identified by Chen et al. through MD simulations in α-Fe: bulk-chain absorption and grain boundary chain absorption models [42]. Radiation resistance mechanisms in nanocrystalline materials are reviewed in more detail elsewhere [43]. These different models attempt to mechanistically rationalize why nanocrystalline materials, with their high grain boundary density, have shown better irradiation tolerance.

On the other hand, some studies have shown that a smaller grain size has no effect on the radiation tolerance or that it can be detrimental under certain conditions. For example, Barr et al. reported an independence of dislocation loop density with grain size in NC-Pt thin films during in situ heavy ion irradiation at 300 °C, showing no improved radiation tolerance with regard to defect density with reduced grain size in the studied NC regime [18]. Chimi et al. observed a larger defect accumulation rate at −258 °C (15 K) under ion irradiation in NC-Au than in CG-Au but a lower rate at room temperature [44]. The authors attribute this behavior to the lower threshold energy for defect production near the grain boundaries at low temperature [44,45]. Moreover, detrimental radiation-induced amorphization at the grain boundaries has been reported in NC-Si during ion irradiation at high temperatures [46]. When grain boundaries absorb interstitials, they leave an excess vacancy concentration in their vicinity, resulting in amorphization. Indeed, nucleation of amorphous Si occurs when the vacancy concentration reaches a critical value.

Experimental studies have shown that smaller grains do not always result in better radiation tolerance depending on the irradiation conditions [18,44,45,46,47]. The work reported by Shen uses an energetical approach to explain the difference in radiation tolerance [48]. With their assessment, there are two opposite effects on the energy of an irradiated material: a smaller grain size (1) results in higher grain boundary energy and (2) decreases the free energy resulting from defects as the defect accumulation in the grain interior is suppressed. In this analysis, the grain size needs to be carefully optimized to balance the two effects [48]. While in theory nanocrystalline materials have high radiation tolerance, the conditions for which nanograins are beneficial to the radiation tolerance are not well understood. Models developed to understand the improved radiation damage only consider simple grain boundary structures under specific irradiation conditions [41,42,43].

While nanocrystalline materials appear very promising in terms of radiation damage tolerance under certain energetic conditions, they suffer from a lack of microstructural stability and they are highly susceptible to grain growth even at low homologous temperatures [29,49]. Irradiation-induced grain growth has been observed in many materials at temperatures as low as −223 °C [9,12,31,32,33,34,35,36], which represents a temperature at which thermally driven grain growth would not be expected. Table 1 summarizes published results for irradiation-induced grain growth in pure Cu. These data are compared to data for pure thin-film NC-Cu without irradiation where purely thermally driven grain growth is observed [27,28]. For this review, Cu was chosen based on the breadth of literature available compared to other FCC materials. While the recommendations offered in Section 8 are applicable to other FCC material systems, summarizing the performance of all FCC materials is beyond the scope of this review.

The studies given in Table 1 show a wide range of test conditions, and fundamentally indicate that outside of the presence of extrinsic stabilizing mechanisms, nanocrystalline Cu grains will grow under most irradiation and thermal conditions, which are often imposed separately. Therefore, a comprehensive understanding of their radiation tolerance under heuristic environments requires nuanced control of the experimental conditions, and more specifically, an understanding of grain growth in environments combining irradiation and thermal effects. The next section will probe these combined environments as it is essential in order to improve the grain structure stability and therefore maintain the high sink density of nanocrystalline materials.

## 3. Grain Growth Regimes in Combined Irradiation/Thermal Environments

**Figure 1:**
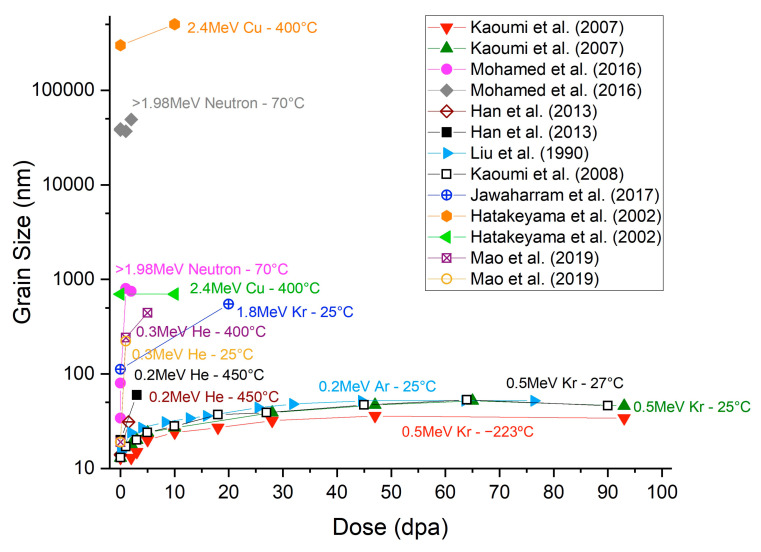
Grain size of irradiated pure Cu as a function of irradiation dose [9,12,22,31,32,34,50,51].

Data from Table 1 are plotted in Figure 1 to indicate the breadth of trends observed in the irradiation of NC-Cu conducted at various temperatures. Some general trends can be deduced from Figure 1. The grain size increases with irradiation dose and grain growth stagnation is observed at high irradiation doses. Additionally, the grain growth rate rises with temperature. Significant contributions from temperature on the grain growth are expected at 400–500 °C, as shown in Table 1 for the unirradiated Cu materials [27,28].

To deconvolute the complex grain growth phenomena caused by combinations of irradiation and thermal exposure, Kaoumi et al. identified three grain growth regimes for nanocrystalline materials under irradiation: (1) a purely thermal regime at temperatures above recrystallization, (2) a thermally assisted regime where both irradiation and thermal effects contribute to the grain growth, and (3) an athermal regime where irradiation effects dominate [31,32]. The first regime has been well covered in the literature [52]. In this regime (thermally activated grain growth), the growth is driven by the reduction in grain boundary free energy and can be described using a power-law-based equation (R^2^ − R_0_^2^ = αt, where R is the mean grain radius, R_0_ the mean initial grain radius, α a temperature-dependent constant, and t is the time) [53,54,55].

In the second (irradiation and thermal effects) and third (primarily irradiation-driven grain growth) regimes, the irradiation–defect interactions come into play. Focusing on the third grain growth regime (irradiation-induced), grain growth has been explained using a thermal spike (e.g., thermal event) approach [32,35,50,56,57]. In this theory, when the collision event ends, the energy of the remaining recoil atoms is thermalized within the lattice, resulting in a localized temperature increase, called a thermal spike. Some studies in the literature use the terminology thermal event to distinguish the thermalized kinetic energy caused by a keV-MeV strike from the thermal spike resulting from predominately electronic energy loss associated with 100 MeV–10 GeV strikes. Liu et al. were the first to suggest thermal spike diffusion phenomenon [50]. If the thermal spike occurs on or near a grain boundary, the atoms are thermally activated and can jump across the boundary [32,57,58,59], resulting in grain boundary migration and thus grain growth.

Similarly to the thermally activated grain growth, power law equations for irradiation-induced grain growth have been developed over the years (equations of the type D^n^ − D_o_^n^ = KΦ, where D is the mean grain diameter, D_o_ the initial mean grain diameter, Φ the ion dose, and K and n are experimental constants) [32,50,60,61]; however, the models do not explain the growth stagnation observed at high irradiation doses [23,30,31,62,63]. The grain growth stagnation has been attributed to the fact that thermal events occur too far from the boundaries to induce boundary motion [35,57,62]. Grain growth only occurs if the cascade volume is larger than the grain volume and overlaps the boundaries [57,62]. In a parallel theory, Singh et al. ascribe grain growth stagnation to the loss of high-mobility grain boundaries during grain growth [63]. However, most irradiation-induced grain growth data have been collected on thin films and an inconvenience of using thin films is the specimen thickness effect [64]. It has been shown that grain growth may stagnate when the grain size approaches the dimension of the film thickness due to surface grooving at the intersections of the boundaries and the film surface [63,64,65].

Modeling and simulation studies have shown that the grain growth kinetics are faster during annealing and irradiation as compared to thermal exposure alone. Using atomistic simulations of a high-angle Σ5(210) grain boundary in a Cu bicrystal, Jin et al. showed that irradiated grain boundaries are about twelve times more mobile than unirradiated boundaries [66]. They surmise this is due to the more frequent rearrangements and migration of atoms. Similarly, MD simulations in NC-Ni comparing thermally and irradiation-induced grain growth have shown that the latter is much faster during the same simulation time (100 ps) [57].

Diffusion plays a significant role in defect annihilation and can partially explain why nanocrystalline metals are theorized to have good radiation tolerance. Smaller grains result in shorter diffusion lengths to nearby sinks, allowing for easier vacancy annihilation at the grain boundaries [67,68]. In larger grains, only vacancies within a certain diffusion distance from the boundaries will migrate and get annihilated, leaving some in the grain interior [67]. Moreover, grain boundaries are known to be “short-circuit” diffusional paths due to their lower atom packing [54]. The diffusivity along grain boundaries increases as the grain size decreases, but also as the misorientation angle increases [3,54]. High-angle grain boundaries typically have lower activation energy for diffusion and therefore higher diffusivities [54]. Grain boundary character as well as the defect cluster size also affect the defect mobility. Atomistic simulations in Cu have demonstrated that mobility decreases as the boundary character complexity and defect cluster size increase [16]. Moreover, irradiation-enhanced diffusivities tend to be much larger than thermal diffusion coefficients (by several orders of magnitude) due to the greater concentration of vacancies and interstitials generated during irradiation [3,69].

Temperature also plays an important role in diffusion. Five material-dependent defect mobility regimes/recovery stages have been defined [1,70,71], with Stage III being the primary regime for the experiments cited in this work. During Stage III, both interstitials and vacancies have enough thermally driven mobility to migrate. Details about the other defect mobility regimes can be found elsewhere [1,70].

While grain growth under combined thermal and irradiation conditions is critical for understanding the evolution of the microstructure and radiation damage tolerance during service, the nature of the grain boundary structure plays a main role in the accommodation of irradiation-induced defects. Considering the grain size alone is not sufficient, it is important to also study grains as a function of distribution, grain boundary character, and chemistry, which will be covered next in Section 4.

## 4. Grain Boundary Character Controlled through Synthesis and Processing

Atomistic simulations in Cu have shown that the interaction between grain boundaries and defects is sensitive to the boundary microstructure [16,21]. Room-temperature heavy ion irradiation of bicrystal Cu shows a higher defect absorption rate in low-angle grain boundaries (LAGBs) due to the cooling-induced lattice strain attracting more point defects [19]. Density functional theory (DFT) calculations conducted on Cu confirm that LAGBs are stronger sinks than high-angle ones [13]. At low angles, the boundary sink strength is high due to the local stress field of the neighboring dislocations, and it increases with the misorientation angle (i.e., higher dislocation density). However, as the misorientation further increases, the dislocation stress fields tend to cancel each other out, decreasing the boundary sink strength [13]. Additionally, Vetterick et al. have shown experimentally and via MD simulations that non-equilibrium grain boundaries are stronger sinks for point defects compared to equilibrium boundaries, due to their higher energy and free volume [72]. In turn, nanocrystalline materials are typically produced by non-equilibrium processes [73], such as severe plastic deformation (SPD) [72,73,74], and thin-film synthesis methods, such as physical vapor deposition (PVD) [72,73]. These approaches have enabled grain boundary engineering attempts to enhance their sink strength [47,51,75,76,77].

As shown in Table 1, most studied nanocrystalline materials are produced via thin-film deposition: sputter deposition [31,32,72,78,79], pulsed deposition [35,80], electrodeposition [34,40], or gas deposition [44,45,81]. Deposited thin films result in high chemical purity materials as they are produced under a clean environment and they typically present columnar grains [82,83]. As discussed previously, an inconvenience of using thin films is the specimen thickness effect, which limits grain growth [63,64,65].

Another way of processing nanomaterials is SPD. It enables production of dense bulk specimens, removing the issue of the specimen thickness effect. However, the smallest grain size achievable by SPD is typically higher than what can be obtained via thin-film deposition. Mechanical milling can produce grain sizes between 5 and 50 nm [84,85]. Similarly, high-pressure torsion (HPT) can achieve grain sizes as low as 10 nm. Equal-channel angular pressing (ECAP) and accumulative roll bonding (ARB), for example, produce ultrafine-grained materials (grain size < 1 μm) [76,86]. Impurities can also be more difficult to control than in thin-film processing due to potential extraction or refining remnants or surface contamination [74,86,87]. Impurities are known to decrease grain boundary mobility due to solute drag [63,88], and can act as grain pinners and retard grain growth [86]. In addition, impurities can trap interstitials and vacancies, delaying the formation of clusters [71]. The published experimental data on irradiation-induced grain growth in Cu have nearly exclusively been obtained from materials processed via thin-film deposition (Table 1), and therefore high-purity specimens. Thus, the impurity effect on the irradiation-induced grain growth cannot be confirmed.

Limited irradiation studies on SPD-processed materials have been reported, with most studies reporting on steels [89]. Nita et al. studied NC-Ni and NC Cu-0.5Al_2_O_3_ processed by ECAP followed by HPT and confirmed that nanograins produced via SPD successfully suppress the irradiation-induced damage [51,77]. Consequently, there is ample opportunity to study the irradiation tolerance of broader nanocrystalline classes of metals produced by SPD methods; however, many caveats must be considered when analyzing the resulting data.

Table 2 compares thin-film deposition and severe plastic deformation in terms of specimen purity, grain size, grain structure, and process scalability. While thin films have some limitations, they allow better control of the grain structure compared to SPD-processed materials. The multitude of techniques available to produce nanocrystalline materials and the processing variables within each synthesis method lead to a lack of consistency, complicating the comparison and analysis of experimental data. For example, when comparing materials with different grain sizes that are processed differently, one should also consider the difference in microstructure (grain boundary misorientation, initial defect density, impurities, etc.).

As Table 2 shows, the grain structure and resulting irradiation tolerance are significantly impacted by the fabrication method. The as-fabricated and irradiated microstructures, however, have mechanical properties that are intricately dependent on the processing and irradiation conditions, as elaborated in Section 5.

## 5. Impact on Mechanical Properties

Irradiating metals at low temperature (<300 °C) usually results in hardening and embrittlement in metals [3,5]. Many studies have reported an increase in yield strength as well as a decrease in uniform elongation with increasing damage level in neutron-irradiated FCC materials [7,34,77,90,91,92,93,94,95,96].

**Figure 2:**
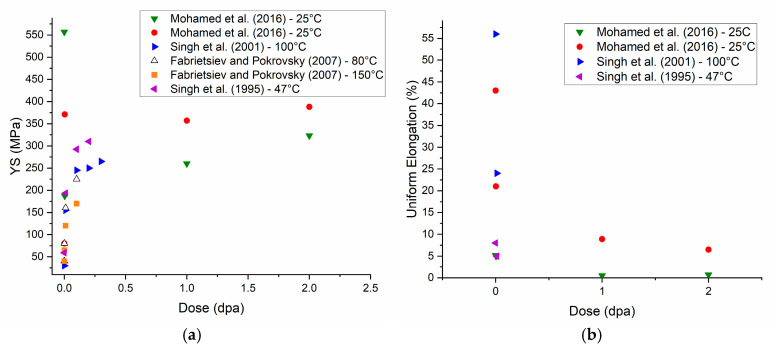
Literature reports of (**a**) 0.2% offset yield strength [34,90,92,96] and (**b**) uniform elongation data for neutron-irradiated Cu as a function of irradiation dose [34,90,96].

Figure 2 compiles yield strength and uniform elongation data from the literature for neutron-irradiated Cu as a function of irradiation dose. Most of the tests have been performed on micro-grained specimens (20–40 μm grain size) and irradiated at low damage levels (<0.5 dpa). Mohamed et al. [34] compared the irradiation-induced hardening in coarse-grained and NC-Cu during neutron irradiation between 0.0034 and 2 dpa. Radiation hardening was observed at all damage levels for the micro-grained material; however, the NC-Cu showed some softening for doses up to 1 dpa, due to irradiation-induced grain growth. Grain size measurements indicate that grain growth saturation occurs above 1 dpa and despite the levelling off of grain size, hardening was observed at 2 dpa [34].

Irradiation hardening has two causes: source hardening and friction hardening [3]. Source hardening is hypothesized to be a result of the irradiation-produced cluster defects providing back stresses on dislocation sources, often modeled by Frank–Read sources, which raise the stress required to enable source multiplication [5,34,96]. Singh et al. [96] as well as Fabrietsiev et al. [91] observed dislocation segments decorated by cluster defects. The unpinning of the defect bound dislocations translates into a yield drop in the tensile curves [91,92,96]. In addition to source hardening, friction hardening is also responsible for the increase in yield strength. Irradiation-produced defects impede the motion of the dislocations [5,34]. The increase in yield strength due to irradiation hardening is proportional to the root square of the number density of obstacles, which is directly proportional to the total fluence. Once the microstructure saturates, the radiation hardening slows down [5]. It is worth noting that the irradiation-induced hardening decreases as the irradiation temperature increases. Fabrietsiev and Pokrovsky compared the properties of Cu irradiated at 80 °C and 150 °C and observed a lower (about 50 MPa) increase in strength between the unirradiated and irradiated conditions for the material irradiated at 150 °C compared to the material irradiated at 80 °C. They ascribe this difference to the higher defect mobility at elevated temperatures; it is easier for the dislocation to overcome obstacles at higher temperatures [92]. Multiple studies have shown that post-irradiation annealing can recover some yield strength [91,96].

In addition to the mechanical properties, both the electrical and thermal conductivities are reduced by the presence of irradiation-induced defects and transmutation products as reported in the case of neutron irradiation [7]. Overall, the effect of irradiation on microstructure and properties is highly dependent on the radiation conditions, and it is important to consider these conditions while studying and comparing irradiation damage in materials. The considerations will be expanded upon in Section 6.

## 6. Impact of Radiation Environment

In addition to the dependence on microstructure, the radiation environment also impacts the observed irradiation damage, which complicates comparisons across experimental reports. Ion irradiation has been used to study radiation damage in materials and emulate neutron irradiation [97]. It is considerably more affordable, enables irradiated material handling, requires shorter cycles [98], and allows better control of the irradiation conditions than neutron irradiation [98,99]. However, the correlation between the two is not straightforward. One main difference between ion and neutron irradiation is the particle energy spectrum: the ion energy spectrum is very narrow, while the one for neutron extends over several orders of magnitude [98]. There is also a large difference in weighted recoil spectra (recoil spectra weighted by number of defects or damage energy produced) between the irradiation species [3,98,99]. Furthermore, the penetration depth is much lower in the case of ion irradiation. While travelling through the lattice, ions undergo electronic excitation (unlike neutrons); they quickly lose energy, resulting in a shorter penetration depth (nm to 100 μm [98,100] compared to greater than 1 mm for neutron irradiation [98,99,100]) and higher damage rate [3,98,99]. This can impact the microstructural evolution [101] and makes the measurement of bulk properties difficult [99]; however, the higher damage rate can be partially compensated for by increasing the irradiation temperature [102,103,104]. For example, to reproduce the effects of neutron irradiation at 300 °C, ion irradiation needs to be conducted at 500 °C [97]; however, the higher ion irradiation temperature can lead to thermal annealing, affecting the microstructure.

Another difference is the type of defects observed in the material after irradiation. Light ions produce isolated damage or small clusters while heavy ions and neutrons create large defect clusters [98,99]. Although heavy ions can reproduce features observed during neutron irradiation, ion irradiation lacks nuclear transmutation products, which can play a significant role in the development of damage [97,100,101,105]. Multiple beam systems have been used to co-implant H and He in addition to heavy ions to more accurately emulate H, He, and knock-on damage production expected in a neutron-irradiated material [97,100,106].

The irradiation species and particle energy will also affect the cascade size and morphology [62,107]. Experimental studies have shown that heavier ions resulted in higher grain growth rate in NC-Ni and NC-Pd [30,62], and from Figure 1 this would appear to be the same in NC-Cu for Kr or Cu ions. The size of the thermal spike/event is also dependent on the recoil energy as well as the target material properties [3,33,36]. Li et al. measured greater grain growth in NC-Au than NC-Pt after room temperature 200 keV Ar^+^ irradiation, and this difference was explained by a lower grain boundary activation energy for Au [62].

Apart from the thermal spike/event caused by the cascade, beam heating can also occur, leading to temperature increases [108]. The heat input is proportional to the beam current; therefore, the beam heating can be limited by limiting beam current. However, this results in longer irradiation times needed to achieve a specific irradiation dose [97]. It is important to note that for the room temperature irradiation-induced grain growth data plotted in Figure 1, the temperature rise from beam heating was negligible [9,31,32,50].

Another important environmental aspect to mention is the mode of irradiation. Irradiation can be conducted using a rastered beam or a broad beam [100]. The raster-scanning mode is considered as pulsed irradiation while the broad beam is steady/continuous irradiation [109]. The irradiation mode affects the material differently due to the different time scales implemented. In the case of pulsed irradiation, during a cycle, a given volume element is under the beam for only a fraction of time. This means the immediate dose rate is much higher than the average one, leading to a high defect production rate. Furthermore, during pulsed irradiation, defects have time to anneal out before the beam passes through again, resulting in lower effective defect production than during continuous irradiation [97]. Experiments have shown that pulsed irradiation suppresses swelling [109,110], but the impact on other microstructural features is less known [100]. In addition, low-frequency (<2 Hz) pulsing can result in local heating, and thus thermal annealing, which limits defect accumulation [109].

## 7. Impact of the Collective Findings on Generating New Knowledge

In the prior sections, we have presented the fundamental mechanisms for grain size stability under irradiation, and the breadth of literature providing reports on these findings. The reports cover a wide span on starting grain sizes and irradiation conditions, many of which do not decouple interlinked thermal and irradiation drivers. These processing and testing variations, in turn, affect the resultant mechanical property findings. Unravelling these findings is not trivial, but some important implications emerge from this review. Firstly, processing definitively impacts the microstructure in ways that affect irradiation damage tolerance. For example, the features of the grain structure, such as grain size distribution, energetics (e.g., LAGBs vs. HAGBs), grain morphologies (equiaxed vs. columnar), and alloy and grain boundary chemistry (thin-film vs. bulk processing), must all be carefully documented and parametrically controlled to reveal valid irradiation grain growth effects under specific irradiation conditions and temperatures. Secondly, the irradiation conditions, such as the type of irradiation (ion, neutron, electron, or others), the applied or generated temperature, the cycle time length, the bombarding species mass, and the beam application (pulsed vs. continuous), all correlate with different energy–materials interactions and thus defect-generation conditions, and therefore must also be carefully controlled within a given measurement. Careful consideration and control of these parameters will allow for the generation and validation of experimental findings, and more confident implementation and validation of computational models. The new knowledge generated from such studies will underpin the design of new materials for nuclear power generation and transmission, such as high-strength, high-conductivity radiation-stable conductors in fusion machines [111].

## 8. Summary and Recommendations

Nanocrystalline materials, with their high sink density, have demonstrated some promise for increased radiation damage tolerance. However, their lack of thermal stability makes them highly prone to grain growth, reducing their sink density and thus their capacity to accommodate irradiation damage.

In this paper, we illuminate how nuance is critical in predicting and understanding grain size stability under irradiation. The large range of radiation environments can lead to significantly different radiation damage, complicating the analysis and comparison of radiation damage effects. In addition, the various processing methods for synthesizing nanocrystalline materials alter the microstructure and therefore the response to irradiation. Notably, grain structure and the impurity content significantly impact the interaction between irradiation defects and sinks.

The extant literature on Cu grain size stability under irradiation reports a range of irradiation conditions and microstructures, complicating one-to-one comparisons and necessitating continued experiments and modelling to advance the understanding of nanostructured materials tailored for use in irradiation environments. We identify multiple thrusts crucial for meaningful comparisons across grain sizes and irradiation conditions:

(a) In-depth material preparation studies to understand the effect of the processing method on the damage tolerance. This includes deeper explorations into bulk processing methods that might be suitable for specific radiation environments. Most irradiation-induced grain growth studies have been conducted on thin-film materials. Studying irradiated bulk materials would allow the effect of impurities to be investigated, as well as the removal of the specimen thickness effect.
(b) Deeper studies of impurity content effects to decipher chemical variations on the damage tolerance, focusing on the difference between lab-grown and commercially processed materials.
(c) Exploratory studies on the interplay of primary knock-on atom (PKA) energy, damage cascade, and irradiation temperature effects.
(d) Higher throughput in situ and ex situ testing to study grain growth effects under a wider span of irradiation doses and/or temperatures on the same starting material such that trends can be reported with higher confidence, at least for the chosen irradiation type (ion vs. neutron vs. electron).
(e) Round-robin type of experiments probing single-sourced Cu samples (with constant range of grain sizes) exposed to the same energy and species to help the community focus on specific irradiation condition effects.

## Figures and Tables

**Table 1:** Select published results for irradiation-induced grain growth for pure Cu.

Purity	Production/ Processing	Irradiation/ Annealing Temperature (°C)	Annealing Time (min)	Radiated Particle	Particle Energy (MeV)	Dose (dpa)	Flux (dpa/s or ion/cm^2^.s)	Initial Grain Size (nm)	Final Grain Size (nm)	Reference
99.99%	Sputter deposition	25	-	He	0.3	1	1.4 × 10^−4^ dpa/s	19	222	[9]
		400		He	0.3	1		19	242	
		400		He	0.3	5		19	422	
NR	Vapor deposition	450	-	He	0.2	3	NR	20	60	[12]
NR	Vapor deposition	450	-	He	0.2	1.5	NR	14	31	[22]
99.999%	Rolled and annealed sheet	400	-	Cu	2.4	10	2.5 × 10^−3^ dpa/s	700	700	[23]
								300,000	500,000	
NR	DC-magnetron sputtering	100	10	-	-	-	-	43	59	[27]
		100	180					43	81	
		100	300					43	104	
		300	60					43	127	
		500	60					43	330	
99.95%	DC-magnetron sputtering	300	60	-	-	-	-	43	77	[28]
		300	180					43	86	
		300	300					43	106	
		500	60					43	175	
		500	180					43	237	
		500	300					43	278	
NR	Sputter deposition	−223	-	Kr	0.5	2	2.5 × 10^16^ dpa/s	13	13	[31]
						3		13	15	
						5		13	20	
						10		13	24	
						18		13	27	
						28		13	32	
						47		13	36	
						93		13	34	
NR	Sputter deposition	25	-	Kr	0.5	2	2.5 × 10^16^ dpa/s	13	18	[31]
						3		13	20	
						5		13	24	
						10		13	27	
						18		13	39	
						28		13	47	
						47		13	52	
						93		13	46	
NR	Sputter deposition	27	-	Kr	0.5	1	2.5 × 10^12^ ions/cm^2^.s	13	17	[32]
						3		13	20	
						5		13	24	
						10		13	28	
						18		13	37	
						27		13	39	
						45		13	47	
						64		13	53	
						90		13	46	
99.999%	Electrodeposition	70–100	-	Neutron	>1.98	0.0034	7.52 × 10^−7^ dpa/s	34	80	[34]
						1		34	800	
						2		34	750	
99.999%	NR	70–100		Neutron	>1.98	0.0034	7.52 × 10^−7^ dpa/s	38,000	39,000	[34]
			-			1		38,000	37,000	
						2		38,000	49,000	
NR	Sputter deposition	25		Ar	0.2	2 *	1.88 × 10^12^, 6.25 × 10^12^, 3.57 × 10^13^ ion/cm^2^.s	15	24	[50]
						4 *		15	27	
						8 *		15	31	
						13 *		15	34	
			-			16 *		15	36	
						25 *		15	44	
						32 *		15	48	
						45 *		15	52	
						64 *		15	52	
						76 *		15	52	
NR	Sputter deposition	25	-	Kr	1.8	20	NR	112	547	[51]

* Calculated from fluence. NR: non-reported.

**Table 2:** Comparison of thin films and SPD-processed bulk materials.

Characteristic	Thin Films	SPD-Processed Bulk Materials
Purity	High purity (vacuum environment, clean surfaces) [82]	Presence of impurities [74,86,87]
Achievable Grain Size	<10 nm [82]	HPT: 10–100 nm [76] Mechanical milling: 5–50 nm [84,85] Other SPD processes: <1 μm [76,86]
Grain Structure	Primarily columnar grains [82,83] Lattice strain from rapid cooling [82]	Equiaxed grains [86] High-angle grain boundaries [86,87]
Grain Structure Control	Grain size can be controlled via substrate temperature [82,83]	Grain boundary misorientation evolves with strain [74] Flash annealing to control grain size
Scalability	Limited by vacuum/clean environment	HPT: size limited (samples typically 10 mm in diameter and 1 mm thick) [76] Other processes: scalable
Limitation	Specimen thickness effect [64]	Difficult to control grain boundary character Impurity level (mechanical milling)

## Data Availability

Not applicable.
